# The Role of Eccentric Strength in 180° Turns in Female Soccer Players

**DOI:** 10.3390/sports5020042

**Published:** 2017-06-17

**Authors:** Paul A. Jones, Christopher Thomas, Thomas Dos’Santos, John J. McMahon, Philip Graham-Smith

**Affiliations:** 1 Directorate of Sport, Exercise & Physiotherapy, University of Salford, Salford, Greater Manchester M6 6PU, UK; c.thomas2@edu.salford.ac.uk (C.T.); t.dossantos@edu.salford.ac.uk (T.D.); j.j.mcmahon@salford.ac.uk (J.J.M.); 2 Aspire Academy, Doha, Qatar; Philip.GrahamSmith@aspire.qa

**Keywords:** change of direction ability, deceleration, velocity, penultimate contact, isokinetic

## Abstract

Previous studies have reported an association between eccentric strength (ECC-STR) and change of direction (COD) ability. Little is known about how ECC-STR facilitates COD maneuvers. The aim of this study was to examine the role of ECC-STR during a 180° COD task in 18 female soccer players. Each player performed six trials of a 180° COD task whereby three-dimensional motion data from 10 Qualisys Pro-Reflex infrared cameras (240 Hz) and ground reaction forces (GRFs) from two AMTI force platforms (1200 Hz) were collected. Relative eccentric knee extensor (ECC-EXT) and flexor (ECC-FLEX) peak torque was collected from both limbs at 60°·s^−1^ using a Kin Com isokinetic dynamometer. Large correlations were revealed between COD performance (time to complete 5 m approach, 180° turn, 5 m return) and ECC-EXT (*R* = −0.674) and ECC-FLEX (*R* = −0.603). Moderate to large correlations were observed between approach velocity (AV) and COD performance (*R* = −0.484) and ECC-EXT (*R* = 0.724). Stronger participants (*n* = 9) recorded significantly (*p* < 0.05) faster AV (4.01 ± 0.18 vs. 3.74 ± 0.24 m·s^−1^, *d* = 1.27) and a greater reduction in velocity (−1.55 ± 0.17 vs. −1.37 ± 0.21 m·s^−1^, *d* = −0.94) during penultimate contact than weaker (*n* = 9) subjects. Greater ECC-STR is associated with faster COD performance in female soccer players, as stronger players are better able to decelerate during penultimate contact from faster approach velocities.

## 1. Introduction

Change of direction (COD) ability or speed has been defined as the ability to decelerate, reverse or change movement direction and accelerate again, and is considered pre-planned [1]. Whilst agility (a rapid and accurate whole-body movement, with change of velocity or direction, in response to a stimulus [2]) is considered highly important in a number of field and court based sports [3], COD ability is an underpinning quality [2] and the development of COD ability is considered important to provide the physical and technical foundation to develop agility [4].

A number of studies have examined the physical determinants of COD ability, revealing associations to linear sprinting ability [1,5], eccentric strength [1,5,6,7], isometric strength [6,7,8,9], concentric strength [6,7], multi-joint dynamic strength [6,7,10], power [11,12] and reactive strength [13,14]. However, due to variations in COD protocols used, muscle strength quality under investigation, methods of assessing a given muscle strength quality, sample population and statistical approaches, conflicting findings have been reported.

The type of muscle strength quality investigated has led to much wide spread confusion as many authors have investigated ‘strength’ in general, without considering that specific strength qualities each may have a role during COD [6]. For instance, during the final contact (turn) of a commonly used 505 test (15 m approach, 180° turn, 5 m return), an athlete will require sufficient eccentric strength to reduce momentum during the braking phase, isometric strength during the plant (amortization) phase and concentric strength during the propulsion phase to help re-accelerate in the opposite direction [6]. Thus, relationships between isolated muscle strength qualities may not always be observed in COD tasks where multiple strength components are required. More investigations examining the role of specific muscle strength qualities in specific phases of COD are necessary.

As mentioned previously, eccentric strength may play a role in reducing momentum during the final stages of approach during a 180° COD task and during the braking phase of final contact. Indeed, several previous studies have reported an association between eccentric strength and 505 COD performance [1,5,6]; and De Hoyo et al. [15] have shown positive benefits of 10 weeks eccentric training on force-time characteristics during side-step and cross cutting in under 19 professional male soccer players. Graham-Smith and Pearson [5] reported a co-efficient of determination of 42.1% between eccentric isokinetic knee extensor strength (ECC-EXT) and 505 test performance in 32 male and female sports students. Similarly, Jones et al. [1] found significant correlations between eccentric isokinetic knee extensor (*R* = −0.529) and flexor strength [ECC-FLEX] (*R* = −0.626) and 505 test performance (2.34 ± 0.12 s) in 38 University sports participants. The authors suggested that eccentric knee extensor strength is important to control knee flexion during final contact when the ground reaction forces (GRFs) acting through the lower limbs are high, whilst eccentric knee flexor (hamstring) strength is important to help generate hip extensor torque to maintain trunk position during deceleration and assist with knee joint stability. Both abovementioned studies also found strong correlations with linear sprinting speed (*R*^2^ = 60–65%). Finally, Spiteri et al. [6] investigated the relationships between a number of lower limb muscle strength qualities and COD performance (505 test and *t*-test) in elite female basketball players. Significant strong correlations (*R* = −0.79 to −0.89) were observed between all specific muscle strength qualities (i.e., dynamic, eccentric, isometric and concentric strength), with eccentric strength identified as the sole predictor of COD performance. One limitation of these studies is that they only examined the association between eccentric strength and global COD performance. No studies to date have examined the role of eccentric strength during COD. It is believed that eccentric strength is important for deceleration in such tasks, but no research has investigated this.

There is a lack of studies that have investigated the association between ‘strength’ and COD mechanics. Previous research [8] compared 45° cutting mechanics between stronger and weaker performers differentiated by performance on a unilateral isometric squat test. The authors found that stronger athletes (*n* = 12) achieved faster COD performance, post stride velocity, greater vertical and horizontal braking forces, vertical propulsive force, vertical braking impulse, horizontal propulsive impulse and angle of peak braking force application than weaker athletes (*n* = 12); suggesting that stronger individuals produce a higher magnitude of plant foot kinetics. However, the study only tested ‘recreational’ team sport athletes comprised of a mixed gender sample. Furthermore, the authors used a controlled approach velocity of 4.5 ± 0.5 m·s^−1^ to control for the effect on GRFs. However, an understanding of the interaction between speed, strength and technique for successful COD performance is required to inform training and conditioning practices to support expressions of agility and COD. Further research [7] examined mechanical and strength differences between faster (*n* = 6) and slower (*n* = 6) elite female basketball players in 505, *t*-test and multidirectional agility tests without controlling approach velocity. Specifically, for the 505 test, the authors found that faster athletes had faster approach velocities, but were not significantly different. Moreover, faster players produced significantly greater vertical braking and propulsion forces, reduced braking and contact times along with greater eccentric and isometric strength. The authors suggested that the greater eccentric strength enabled faster athletes to complete the direction change with a shorter braking time through application of greater braking force (due to greater eccentric strength) allowing a faster transition into the propulsion phase [7]. However, this study only examined vertical GRFs. Furthermore, in the above-mentioned studies, Spiteri et al. [7,8] only examined final foot contact, whereas recent studies have highlighted the role of penultimate contact during cutting and pivoting (180° turns) for injury prevention [16] and performance [17,18]. Thus, in order to evaluate the association between strength, speed and technique in COD, an examination of penultimate contact is required. 

The aim of the study was to examine the role of eccentric strength during performance of a 180° COD task in female soccer players. In order to achieve this aim, the study had the following objectives; (1) to explore the relationships between COD performance (completion times), ECC-EXT and ECC-FLEX and approach velocity, (2) examine the differences between stronger and weaker players in regard to deceleration into the turn (i.e., penultimate and final contact of the turn), and (3) explore the kinetic (i.e., GRFs and hip and knee joint moments) differences between stronger and weaker players during weight acceptance (braking phase) of penultimate and final contact. It was hypothesized that there is an association between eccentric strength, approach velocity and COD performance and that stronger players produce faster COD performance times, through a faster approach velocity and greater deceleration during the final two contacts of the task.

## 2. Materials and Methods

Eighteen female soccer players participated in the study. The mean ± SD age, height and mass was 21.6 ± 4.3 years, 1.67 ± 0.07 m and 60.3 ± 6.3 kg, respectively. All players played in the top two tiers of English Women’s football at the time of the study. For inclusion in the study, all players needed to have played soccer for a minimum of 5 years and regularly performed one game and two structured skill-based sessions per week. Sixteen of the 18 players described themselves as right leg dominant. All players were free from injury during the course of the study and none of the players had suffered prior traumatic knee injury such as anterior cruciate ligament injury. Data collection took place during the players’ pre-season. Approval for the study was provided by the University of Salford Ethics committee (ethics approval code: HSCR 16-03). All subjects provided written informed consent prior to participating in the study. 

Each participant attended the lab on three separate occasions. The first occasion was a familiarization session across the protocols used in the study with data collected on the subsequent sessions. The first of these sessions involved participants performing multiple trials of a 180° COD task, whilst collecting three-dimensional motion and force data. The second session involved isokinetic assessment of knee extensor and flexor strength.

**Figure 1:**
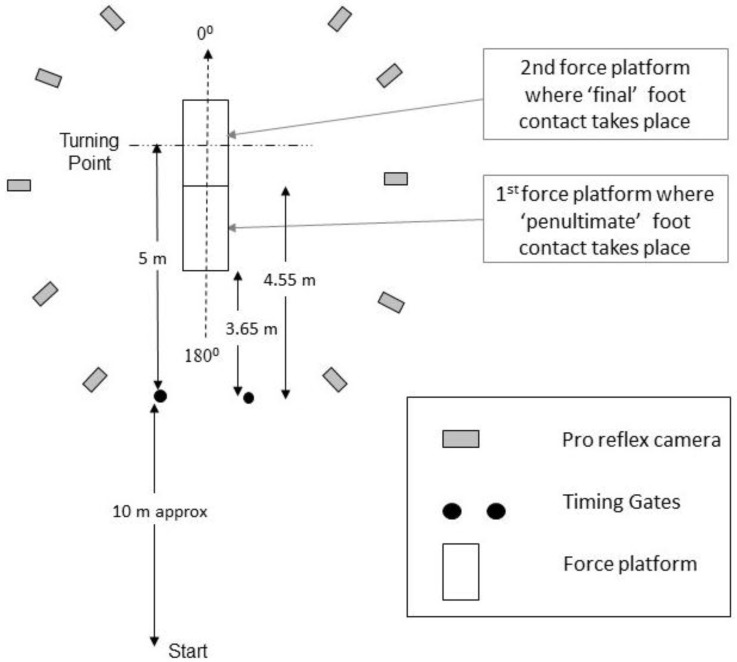
A plan view of the experimental set-up for the change of direction task.

Testing took place on an indoor Mondo running surface. The COD task involved the subjects running towards two force platforms. The first force platform was used to measure GRFs from the penultimate foot contact, whilst the second force platform was used to measure GRFs from the final foot contact (Figure 1). Prior to the turn, the subject ran through a set of timing lights positioned 5 m from the center of the last platform. The subjects then turned (180°) back to the original starting position once contacting the end force platform with their preferred leg (the leg they preferred to turn off). Total time to complete the task was measured using a set of Brower timing lights (Draper, UT). The timing lights were set at approximate hip height for all subjects as previously recommended [19], to ensure that only one body part (i.e., lower torso) broke the beam. Task completion time was used as a global performance measure. Each subject started approximately ≤10 m behind the first set of timing lights similar to a 505 test. Some flexibility was allowed for the exact starting point of each subject to allow for the subjects’ differing stride pattern as they approached the end two force platforms. Each subject was allowed time prior to data collection to identify their exact starting point to ensure appropriate force platform contacts. 

During data collection, all subjects performed a minimum of six ‘good’ trials of the COD task. A good trial was considered to involve; (1) a straight approach to the force plates without prior stuttering or prematurely turning prior to final contact, (2) contact with the first force platform during penultimate foot contact and (3) contact with the central portion of the last platform during final contact to ensure a homogeneous distance of travel between trials. Trials were subsequently disqualified if the subject did not adhere to these characteristics. Verbal feedback was provided to rectify any of the abovementioned aspects on subsequent trials. The fastest three trials were used for analysis.

The following procedures have been reported previously [16]. Thus, only a brief overview is provided here. Reflective markers (14 mm spheres) were placed on body landmarks [16] of each subject by the same researcher to ensure marker placement consistency. Subjects wore ‘cluster sets’ (four reflective markers attached to a light weight rigid plastic shell) attached using Velcro elasticated wraps on the right and left thigh and shin to approximate the motion of these segments during dynamic trials. The pelvis and trunk cluster sets were attached using an elasticated belt and Lycra ‘crop top’, respectively. 

Three-dimensional motions of these markers were collected whilst performing each trial using 10 Qualisys ‘Pro reflex’ (Model no. MCU 240) infrared cameras (240 Hz) operating through Qualisys Track Manager software (version 1.10.282) (Qualisys, Gothenburg, Sweden). GRFs were collected from two AMTI (Advanced Mechanical Technology, Inc., Watertown, MA, USA) (Model no. 600900) force platforms (1200 Hz) embedded into the running track. The force platform arrangement allowed data to be collected for both the final and penultimate contact. 

From a standing trial, a 6-degree-of-freedom model of the lower extremity and trunk was created for each participant, including trunk, pelvis, thigh, shank and foot using Visual 3D software (C-motion, version 3.90.21). This kinematic model was used to quantify the motion at the hip, knee and ankle joints using Cardan angle sequence [20]. The local coordinate system was defined at the proximal joint center for each segment. The static trial position was designated as the subject’s neutral (anatomical zero) alignment, and subsequent kinematic measures were related back to this position. Lower limb joint moments were calculated using an inverse dynamics approach [21] through visual 3D software (C-motion, version 3.90.21) and are defined as internal moments. Segmental inertial characteristics were estimated for each participant [22]. The model utilized a CODA pelvis orientation [23] to define the location of the hip joint center. The knee and ankle joint centers were defined as the mid-point of the line between lateral and medial markers. The trials were time normalized for each subject, with respect to the ground contact time of the COD task. Initial contact was defined as the instant after ground contact when the vertical GRF (vGRF) was higher than 20 N and end of contact was defined as the point when the vGRF subsided past 20 N for both penultimate and final contacts. The weight acceptance phase of ground contact was defined as from the instant of initial contact (vGRF >20 N) to the point of maximum knee flexion during ground contact as used previously [16,24]. Joint coordinate and force data were smoothed in visual 3D with a Butterworth low pass digital filter with cut-off frequencies of 12 Hz and 25 Hz, respectively. Cut off frequencies were selected based on a residual analysis [21] and visual inspection of the data. 

Trunk and lower limbs’ center of mass (model CM) was computed as recommended by Vanrentergehm et al. [25] to evaluate approach velocity. Horizontal model CM position was determined from 10 frames prior to penultimate contact to 10 frames from the toe-off of the final contact. The first derivative of the model CM position was computed to derive horizontal (x) velocity over this period. Horizontal velocity at the start of penultimate contact was determined to represent the approach velocity of the participant for that trial. In addition, horizontal velocity at the start of final foot contact and the end of the weight acceptance phase of final contact was determined for each trial. This allowed an evaluation of the change in velocity (deceleration) during the final two contacts, including (1) change in velocity from start of penultimate contact to final contact (ΔPEN), (2) start of penultimate contact to end of weight acceptance of final contact (ΔPEN-FIN) and (3) start of final contact to end of weight acceptance of final contact (ΔFIN). During the weight acceptance phase of penultimate and final contacts of the COD task, peak and average vertical (Fz) and horizontal (anterior–posterior) (Fx) GRFs were determined along with peak sagittal plane knee and hip moments.

Gravity-corrected isokinetic peak torque from right and left knee flexor and extensor muscle groups was assessed at 60°·s^−1^ in eccentric modes using a Kin Com (Chattanooga Group, Tennessee) isokinetic dynamometer using similar methods reported previously [26]. The dynamometer was calibrated according to manufacturers’ standardized procedures. The subjects were seated with the hip joint at 90° (supine position = 0°). The axis of rotation of the dynamometer shaft was aligned with the best approximation of the knee joint axis of rotation, midway between the lateral condyles of the femur and tibia. The cuff of the dynamometer lever arm was attached to the ankle, just proximal to the malleoli. Extraneous movement was prevented by straps, positioned at the hip, shoulders and tested thigh. Subjects were instructed to hold onto the handles located underneath the seat. Range of motion was set as close to 90° as possible (0° = full knee extension).

Eight sub-maximal concentric knee extension and flexion movements were performed as a warm-up. This followed 3 minutes of stationary cycling (60 rpm) on a cycle ergometer (Wattbike Ltd., Nottingham, UK). The ‘overlay’ method was selected for data collection to generate individual angle–torque graphs per repetition. The repetition exhibiting the highest torque from four maximal efforts for each muscle group (10 s recovery between efforts) was saved for further analysis [27]. Data were exported in ASCII format into Microsoft Excel for further examination. Phases of acceleration and deceleration, using a tolerance of ±1°·s^−1^, were deleted from the analysis. Right and left eccentric peak torque values were normalized by body mass and averaged across limbs for both muscle groups (ECC-EXT, ECC-FEX) and subsequently used for statistical analysis.

Statistical analysis was performed in SPSS for Windows (version 23, IBM, New York, NY, USA). Normality of all data was inspected using a Shapiro–Wilks test. To explore relationships between eccentric strength (knee extensors and flexors), approach velocity and COD performance time, Pearson’s product moment correlation was performed for normally distributed variables. From this, co-efficients of determination (*R*^2^ × 100) were also calculated. Spearman’s rank correlation was performed on non-normally distributed variables. Significance level for Pearson’s (R) and Spearman’s (ρ) correlation were Bonferroni corrected to reduce likelihood of type 1 error. Correlations were evaluated as follows: trivial (0.0–0.09), small (0.10–0.29), moderate (0.30–0.49), large (0.50–0.69), very large (0.70–0.89), nearly perfect (0.90–0.99), and perfect (1.0) [28]. In addition, subjects were separated into stronger and weaker groups based on relative eccentric peak torque for the knee extensors and flexors. Subjects above the 50th percentile were assigned to the ‘strong’ group and those below the 50th percentile were assigned into the ‘weak’ group similar to previous research [7,8]). In allocating these groups, two players swapped groups from examining ECC-EXT to then examining ECC-FLEX. Paired samples *t*-tests were performed for normally distributed data, whilst Mann–Whitney U tests were performed for non-normally distributed data to compare differences between groups in terms of performance times and approach velocity. A Levene’s test was used to inspect the data for equality of variances with appropriate adjustments (equality of variances not assumed) for violation of this assumption. Effect sizes were calculated using Cohen d (mean strong group–mean weak group/SD pooled) and interpreted as trivial (<0.19), small (0.20–0.59), moderate (0.60–1.19), large (1.20–1.99), and very large (2.0–4.0) [29]. Moreover, to provide a greater understanding of the role of eccentric strength in decelerating during the COD task, similar comparisons were made between groups for velocity, GRFs and knee and hip joint moments.

## 3. Results

Normality was confirmed for all COD performance times, strength variables and approach velocity. Mean ± SD COD performance times, ECC-EXT, ECC-FLEX, and approach velocities were 2.67 ± 0.12 s, 3.36 ± 0.55 Nm·kg^−1^, 1.63 ± 0.3 Nm·kg^−1^, 3.88 ± 0.25 m·s^−1^, respectively.

### 3.1. Relationships between COD Performance, Eccentric Strength and Approach Velocity

**Figure 2:**
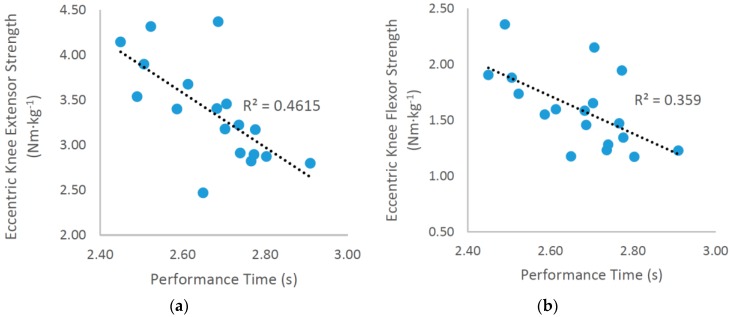
Scatter diagrams illustrating the relationships between (**a**) change of direction (COD) performance (time) and relative eccentric knee extensor peak torque (average of both limbs); (**b**) COD performance (time) and relative eccentric knee flexor peak torque (average of both limbs).

- Significant large correlations were revealed between relative ECC-EXT strength (*R* = −0.674, *R*^2^ = 45%, P < 0.01) and relative ECC-FLEX strength (*R* = −0.603, *R*^2^ = 36%, *p* < 0.05) and COD performance time (Figure 2).
- A moderate correlation was observed between approach velocity and COD performance time (*R* = −0.484, *R*^2^ = 23%). In addition, a significant very large correlation was revealed between relative ECC-EXT strength and approach velocity (*R* = 0.724, *R*^2^ = 52%, *p* < 0.01).
- Incidentally, moderate correlations were found between ΔPEN (ρ = −0.562) and ΔPEN-FIN (*R* = −0.443) with ECC-EXT, illustrating an association between eccentric knee extensor strength and deceleration ability.

### 3.2. Velocity Profle Differences between Stronger and Weaker Participants

Table 1 compares velocity profile data between stronger and weaker subjects.

- For ECC-EXT, significant differences (*p* < 0.05) with moderate to very large effect sizes were observed between performance times, approach velocity and ΔPEN between stronger and weaker participants (Table 1).
- For ECC-FLEX, no significant differences (*p* > 0.05) were observed between stronger and weaker participants for performance times and velocity profile variables (Table S1). However, moderate effect sizes were reported for performance times (strong 2.63 ± 0.12 s; weak 2.71 ± 0.13 s; *d* = −0.64), approach velocity (strong 3.96 ± 0.21 m·s^−1^; weak 3.79 ± 0.28 m·s^−1^; *d* = 0.69), ΔPEN (strong −1.52 ± 0.16 m·s^−1^; weak −1.40 ± 0.24 m·s^−1^; *d* = −0.59) and ΔPEN-FIN (strong −3.72 ± 0.23 m·s^−1^; weak 3.50 ± 0.36 m·s^−1^; *d* = −0.73).

### 3.3. Kinetic Differences between Stronger and Weaker Participants

Kinetic differences between stronger and weaker participants with regard to ECC-EXT are presented in Table 2.

- Significant differences (*p* < 0.05) with large effect sizes were found for average horizontal GRF during penultimate contact, average horizontal GRF and vertical GRF during final contact (Table 2). Moderate effect sizes (*d* > −0.95) were also found for peak horizontal GRF and peak hip extensor moments during penultimate contact.
- For ECC-FLEX, no significant differences (*p* > 0.05) were observed between stronger and weaker participants for GRFs or joint moments during final and penultimate contact (Table S2). However, a moderate effect size was found for average horizontal GRF during final contact between stronger and weaker subjects (strong −0.95 ± 0.10 bw; weak −0.84 ± 0.15 bw; *d* = −0.86).

## 4. Discussion

The aim of the study was to examine the role of eccentric strength during performance of a 180° COD task in female soccer players. To achieve this aim, the study had the following objectives: (1) to explore the relationships between COD performance (completion times), ECC-EXT and ECC-FLEX and approach velocity; (2) examine the differences between stronger and weaker players in regard to deceleration into the turn and (3) explore the kinetic differences between stronger and weaker players during weight acceptance of penultimate and final contact. The results of the study revealed large correlations between COD performance and eccentric knee extensor and flexor strength (*R* > −0.603). In addition, moderate to large correlations were observed between approach velocity and COD performance (*R* = −0.484) and ECC-EXT (*R* = 0.724). Stronger participants (*n* = 9) recorded significantly (*p* < 0.05) faster approach velocities (*d* = 1.27) and a greater reduction in velocity (*d* = −0.94) during penultimate contact than weaker (*n* = 9) subjects. Therefore, it appears that greater eccentric strength is associated with faster COD performance in female soccer players. Furthermore, players with greater ECC-EXT strength are better able to decelerate during penultimate contact from faster approach velocities, substantiating the study’s hypotheses.

The primary finding that eccentric strength is associated with COD performance substantiates previous findings [1,5,6,7] and highlights the importance of eccentric strength in COD tasks that require substantial braking prior to initiating a direction change. Previous studies [1,5,6,7] have only examined the relationships between eccentric strength and global performance measures (i.e., 505 time). The present study is the first to examine the role of eccentric strength during deceleration prior to COD. The moderate to large correlations observed between approach velocity (velocity at the start of penultimate contact) and COD performance and eccentric strength may suggest that there is an interaction between speed and strength in delivering faster COD performance. Athletes with greater eccentric strength are able to approach faster as they are better able to tolerate the greater GRF’s associated with a faster approach. Furthermore, moderate correlations (*R* > 0.44) were observed between ECC-EXT and the magnitude of decline in velocity during penultimate contact and during penultimate contact to the end of the weight acceptance phase of final contact; and significant differences (*p* < 0.05) were observed between stronger and weaker athletes (relative eccentric knee extensor strength) with regard to approach velocity and the decline in velocity during final contact. These results further support the idea that eccentrically stronger athletes are better at decelerating during the final stages of the approach and thus, can approach with a faster velocity perhaps due to a ‘self-regulation’ effect (i.e., a player approaches faster based on the deceleration load they know/feel they can tolerate), which can lead to faster overall COD performance. 

Interestingly, examination of the velocity profile data and the significant difference between stronger and weaker athletes in terms of the magnitude of decline in horizontal velocity during penultimate contact also highlights the importance of penultimate contact during deceleration. This is supported by previous research that has shown an association between penultimate contact braking forces and COD performance [17,18]. Graham-Smith et al. [17] found a significant association between peak horizontal braking forces during penultimate contact and performance times (*R* = −0.674, *R*^2^ = 45.4%); whilst Dos’Santos et al. [18] found an association between greater horizontal braking forces during penultimate contact (*R* = −0.337) and horizontal braking force ratio [peak horizontal braking force final/peak horizontal braking force penultimate] (*R* = −0.429) with COD performance and that faster athletes were significantly lower with regard to the latter (i.e., produce greater force in penultimate relative to final contact) in similar 180° COD tasks. Together, it might be suggested that penultimate contact or prior steps are pivotal in the interaction between strength, speed and technique with regard to COD performance.

The kinetic comparisons between stronger and weaker athletes provide further substance to the role of penultimate contact. The present study found significant differences with large effects sizes between stronger and weaker athletes in terms average horizontal GRF during penultimate contact and moderate effect size (*d* > −0.95) differences for peak horizontal GRF during penultimate contact and peak hip extensor moments during penultimate contact. Thus, the eccentrically stronger athletes are producing greater braking force during penultimate contact to allow greater deceleration from a faster approach. Significant differences with large effect sizes were also observed for average horizontal GRF and vertical GRF during final contact. This supports previous findings by Spiteri et al. [8] that stronger athletes produce greater vertical and horizontal braking forces during final contact, which facilitates faster COD performance. Moreover, Spiteri et al. [7] also found that elite basketball players that were faster during a 505 test, were significantly stronger both in eccentric and isometric tests and were able to produce greater vertical braking forces during final contact, which enabled a shorter braking time and a faster transition into the propulsion phase. The results of the present study provide further support for the importance of eccentric strength to allow a more efficient braking phase during final contact.

The study revealed stronger correlations for ECC-EXT with COD performance than ECC-FLEX, contrary to Jones et al. [1]. Greater eccentric knee flexor (hamstring) strength may assist in helping to generate hip extensor torque during penultimate and final contact to control trunk flexion during these phases as well as providing hamstring co-contraction to assist with knee joint stability during the final contact. No significant differences (*p* > 0.05) were observed between stronger and weaker players in terms of ECC-FLEX for performance times, velocity profile variables and kinetic characteristics. Moderate effect sizes were reported for performance times (*d* = −0.64), approach velocity (*d* = 0.69), ΔPEN (*d* = −0.59) and ΔPEN-FIN (*d* = −0.73) and average horizontal GRF during final contact (*d* = −0.86) between stronger and weaker players with regard to ECC-FLEX, thus, the results of the study suggest that ECC-FLEX may have a minor role in assisting with deceleration mechanics during such COD tasks.

A limitation of the present study was the relatively low sample size to compare strong and weak players, thus, future studies should continue to examine the role of specific muscle strength qualities in COD with greater samples of sports players. Furthermore, the findings of the study may only pertain to a 180° COD task. Therefore, future studies should examine the associations between specific muscle strength qualities in specific phases of other common COD tasks such as cutting. Another limitation of the present study was the use of isokinetic assessment to quantify eccentric strength, which may be considered to have little transfer to dynamic multi-joint movements. Isokinetic methods were used in the present study as this approach has been used in previous research [1,5], was considered reliable [26] and a safe method to use with this population of athletes. Previous research [6,7] has assessed eccentric strength through an eccentric only back squat and perhaps similar future research should be performed using more functional strength assessments. Furthermore, the present study only assessed COD performance turning with the limb the subjects preferred to turn off and thus, no further comparison could be made between turning with either limb. Future research should examine the interaction between CODs from either limb and between limb strength differences to allow a greater understanding of how limb preference or bilateral strength imbalance impact the deceleration strategy turning with either limb. 

Although the results of the present study highlight the importance of eccentric strength for COD performance, a cause–effect relationship cannot be deduced. De Hoyo et al. [15] investigated the effects of 10 weeks eccentric over-load training (eccentric flywheel device) on kinetic parameters during cross-over (45°) to side-step (60°) cutting in Under 19 professional soccer players. Between group analysis revealed that eccentric training led to substantial improvements in contact time, time spent braking during side-step cutting, and relative peak braking force and impulse during cross-cutting. Thus, eccentric strength training may indeed be beneficial in improving COD performance. However, more research is required to examine the impact of eccentric strength training on performance and deceleration kinematics and kinetics during a 180° COD task.

## 5. Conclusions

The present study examined the role of eccentric strength in performance of a 180° COD task in high level female soccer players. The study found large associations between eccentric knee extensor and flexor strength and COD performance, as well as moderate associations between approach velocity and COD performance and ECC-EXT, suggesting that COD performance involves a complex interaction between strength, speed and technique. Eccentrically stronger athletes had significantly faster performance times, approach velocities and greater decline in horizontal velocity during penultimate contact, highlighting the importance of eccentric strength in deceleration during such tasks. Stronger athletes produced greater average horizontal braking forces during penultimate contact, suggesting that penultimate contact is pivotal in the interaction between strength, speed and technique for successful change of direction performance.

## Figures and Tables

**Table 1:** Differences in performance time and velocity profile variables between stronger and weaker subjects based on relative eccentric knee extensor scores.

Variable	Strong (*n* = 9)	Weak (*n* = 9)	*p*	*d*
Performance Time (s)	2.58 ± 0.10	2.76 ± 0.07	<0.0001	−2.09
Eccentric Knee Extensor Peak Torque (Nm·kg^−1^)	3.80 ± 0.39	2.93 ± 0.24	<0.0001	2.69
Approach velocity (m·s^−1^) ^1^	4.01 ± 0.18	3.74 ± 0.24	0.015	1.27
ΔPEN (m·s^−1^) ^2^	−1.55 ± 0.17	−1.37 ± 0.21	0.034 ^5^	−0.94
ΔFIN (m·s^−1^) ^3^	−2.16 ± 0.19	−2.14 ± 0.3	0.919	−0.08
ΔPEN-FIN (m·s^−1^) ^4^	−3.71 ± 0.22	−3.51 ± 0.37	0.189	−0.66

^1^ Approach velocity is defined as the horizontal model center of mass velocity at the start of penultimate contact. ^2^ change in velocity from start of penultimate contact to start of final contact. ^3^ Change in horizontal velocity from start of final contact to end of weight acceptance phase of final contact. ^4^ Change in velocity from start of penultimate contact to end of weight acceptance phase of final contact. ^5^ Mann–Whitney U test performed.

**Table 2:** Kinetic differences between stronger and weaker subjects based on relative eccentric knee extensor scores.

Variable	Strong (*n* = 9)	Weak (*n* = 9)	*p*	*d*
Ground Reaction Forces				
Peak VGRF during Penultimate contact (bw)	3.44 ± 0.74	3.05 ± 0.45	0.190	0.64
Ave VGRF during Penultimate contact (bw)	0.78 ± 0.09	0.76 ± 0.05	0.518	0.27
Peak HGRF during Penultimate contact (bw)	−2.16 ± 0.37	−1.77 ± 0.41	0.052	−1.00
Ave HGRF during Penultimate contact (bw)	−0.53 ± 0.06	−0.45 ± 0.07	0.009	−1.23
Peak VGRF during Final contact (bw)	3.03 ± 0.45	2.76 ± 0.70	0.354	0.46
Ave VGRF during Final contact (bw)	1.38 ± 0.09	1.25 ± 0.11	0.011	1.29
Peak HGRF during Final contact (bw)	−2.04 ± 0.23	−1.75 ± 0.44	0.107	−0.83
Ave HGRF during Final contact (bw)	−0.99 ± 0.06	−0.79 ± 0.11	<0.0001	−2.26
Joint Moments				
Penultimate Hip Extensor Moment (Nm·kg^−1^)	−3.57 ± 0.78	−2.90 ± 0.62	0.060	−0.95
Penultimate Knee Extensor Moment (Nm·kg^−1^)	3.15 ± 0.55	3.08 ± 0.37	0.783	0.15
Final Hip Extensor Moment (Nm·kg^−1^)	−2.85 ± 0.74	−2.56 ± 0.78	0.432	−0.38
Final Knee Extensor Moment (Nm·kg^−1^)	2.25 ± 0.42	2.29 ± 0.20	0.817	0.12

Ave = average, VGRF = Vertical GRF, HGRF = Horizontal GRF.

## Supplementary Materials

The following are available online at http://www.mdpi.com/2075-4663/5/2/42/s1, Table S1: Differences in performance time and velocity profile variables between stronger and weaker subjects based on relative eccentric knee flexor scores. Table S2: Kinetic differences between stronger and weaker subjects based on relative eccentric knee flexor scores.
