# Supplementary material for: The Role of Eccentric Strength in 180° Turns in Female Soccer Players
Source: Sports (Basel). 2017 Jun 17;5(2):42. doi: 10.3390/sports5020042 (PMC5968983; doi:10.3390/sports5020042)
Supplement: Supplementary file 1 [file sports-05-00042-s001.pdf]

Supplementary Materials: The Role of Eccentric Strength in 180° Turns in Female Soccer Players

Paul A. Jones, Christopher Thomas, Thomas Dos'Santos, John J. McMahon and Philip Graham-Smith

Table S1. Differences in performance time and velocity profile variables between stronger and weaker subjects based on relative eccentric knee flexor scores.

Variable	Strong (*n* = 9)	Weak (*n* = 9)	*p*	*d*
Performance Time (s)	2.63 ± 0.12	2.71 ± 0.13	0.247	-0.64
Eccentric Knee Flexor Peak Torque (Nm·kg^{-1})	1.87 ± 0.21	1.40 ± 0.14	<0.001	2.63
Approach velocity (m·s^{-1})[1]	3.96 ± 0.21	3.79 ± 0.28	0.732	0.69
ΔPEN (m·s^{-1})[2]	−1.52 ± 0.16	−1.40 ± 0.24	0.269	−0.59
ΔFIN (m·s^{-1})[3]	−2.20 ± 0.18	−2.10 ± 0.29	0.233	−0.41
ΔPEN-FIN (m·s^{-1})[4]	−3.72 ± 0.23	−3.50 ± 0.36	0.137	−0.73

[1]Approach velocity is defined as the horizontal model center of mass velocity at the start of penultimate contact. [2]change in velocity from start of penultimate contact to start of final contact. [3]Change in horizontal velocity from start of final contact to end of weight acceptance phase of final contact. [4]Change in velocity from start of penultimate contact to end of weight acceptance phase of final contact.

Table S2. Kinetic differences between stronger and weaker subjects based on relative eccentric knee flexor scores.

Variable	Strong (*n* = 9)	Weak (*n* = 9)	*p*	*d*
Ground Reaction Forces				
Peak VGRF during Penultimate contact (bw)	3.40 ± 0.60	3.09 ± 0.65	0.308	0.50
Ave VGRF during Penultimate contact (bw)	0.76 ± 0.09	0.78 ± 0.06	0.199	−0.26
Peak HGRF during Penultimate contact (bw)	−2.05 ± 0.35	−1.89 ± 0.51	0.441	−0.37
Ave HGRF during Penultimate contact (bw)	−0.50 ± 0.08	−0.48 ± 0.08	0.534	−0.25
Peak VGRF during Final contact (bw)	3.01 ± 0.44	2.78 ± 0.72	0.433	0.39
Ave VGRF during Final contact (bw)	1.35 ± 0.09	1.28 ± 0.14	0.170	0.59
Peak HGRF during Final contact (bw)	−2.00 ± 0.24	−1.80 ± 0.46	0.452	−0.55
Ave HGRF during Final contact (bw)	−0.95 ± 0.10	−0.84 ± 0.15	0.085	−0.86
Joint Moments				
Penultimate Hip Extensor Moment (Nm·kg^{-1})	−3.42 ± 0.81	−3.06 ± 0.71	0.321	−0.47
Penultimate Knee Extensor Moment (Nm·kg^{-1})	3.20 ± 0.56	3.03 ± 0.33	0.440	0.37
Final Hip Extensor Moment (Nm·kg^{-1})	−2.82 ± 0.69	−2.59 ± 0.84	0.523	−0.30
Final Knee Extensor Moment (Nm·kg^{-1})	2.19 ± 0.40	2.35 ± 0.20	0.294	−0.51

Ave = average, VGRF = Vertical GRF, HGRF = Horizontal GRF.
